# *Cronobacter sakazakii* Infections in Two Infants Linked to Powdered Infant Formula and Breast Pump Equipment — United States, 2021 and 2022

**DOI:** 10.15585/mmwr.mm7209a2

**Published:** 2023-03-03

**Authors:** Julia C. Haston, Shanna Miko, Jennifer R. Cope, Haley McKeel, Cynney Walters, Lavin A. Joseph, Taylor Griswold, Lee S. Katz, Ashley A. Andújar, Laura Tourdot, Joshua Rounds, Paula Vagnone, Carlota Medus, JoAnn Harris, Robert Geist, Daniel Neises, Ashley Wiggington, Trey Smith, Monica S. Im, Courtney Wheeler, Peyton Smith, Heather A. Carleton, Christine C. Lee

**Affiliations:** ^1^Division of Foodborne, Waterborne, and Environmental Diseases, National Center for Emerging and Zoonotic Infectious Diseases, CDC; ^2^Epidemic Intelligence Service, CDC; ^3^Minnesota Department of Health; ^4^Hospital B; ^5^Kansas Department of Health and Environment; ^6^Missouri Department of Health and Senior Services; ^7^Laboratory Leadership Service, CDC.

*Cronobacter sakazakii,* a species of gram-negative bacteria belonging to the Enterobacteriaceae family, is known to cause severe and often fatal meningitis and sepsis in young infants. *C. sakazakii* is ubiquitous in the environment, and most reported infant cases have been attributed to contaminated powdered infant formula (powdered formula) or breast milk that was expressed using contaminated breast pump equipment (*1*–*3*). Previous investigations of cases and outbreaks have identified *C. sakazakii* in opened powdered formula, breast pump parts, environmental surfaces in the home, and, rarely, in unopened powdered formula and formula manufacturing facilities (*2*,*4*–*6*). This report describes two infants with *C. sakazakii* meningitis reported to CDC in September 2021 and February 2022. CDC used whole genome sequencing (WGS) analysis to link one case to contaminated opened powdered formula from the patient’s home and the other to contaminated breast pump equipment. These cases highlight the importance of expanding awareness about *C. sakazakii* infections in infants, safe preparation and storage of powdered formula, proper cleaning and sanitizing of breast pump equipment, and using WGS as a tool for *C. sakazakii* investigations.

In September 2021, a state public health department reported an infant with *C. sakazakii* infection to CDC (patient A). In February 2022, a physician reported another case to CDC and a different state health department (patient B). CDC was invited to participate in the investigations and distributed case report forms to obtain detailed feeding information about the two cases. Patient isolates and environmental samples were sent to CDC, state public health laboratories, and other federal agencies for isolation, identification, and WGS analysis of *C. sakazakii*.

The first case occurred in September 2021 in a full-term (gestation of 40 weeks, 1 day) male infant (patient A) born to a healthy mother who had an uncomplicated pregnancy and spontaneous vaginal delivery. At age 14 days, the infant was evaluated at hospital A for fever, irritability, and excessive crying, oral candidiasis (thrush), and diaper dermatitis. Before his illness, he was fed both expressed breast milk and powdered formula. A lumbar puncture was performed, and *C. sakazakii* was isolated from the cerebrospinal fluid (CSF). The infant was admitted to the hospital and treated with intravenous antibiotics for 21 days; he made a full recovery with no apparent long-term sequelae (Table).

**TABLE:** Patient characteristics and testing results in two infant cases of *Cronobacter sakazakii* infection — United States, 2021 and 2022

Characteristic	Patient A (2021)	Patient B (2022)
Age at illness onset	14 days	20 days
Gestational age at birth	40 wks 1 day	30 wks 6 days
Admitting hospital	Hospital A	Hospital B
Feeding source	Powdered infant formula and expressed maternal milk, by bottle	Expressed maternal milk with added liquid human milk fortifier, by orogastric tube
Disease manifestation	Meningitis	Meningitis and bacteremia
Outcome	Survived	Died
Environmental samples positive for *C. sakazakii*	Opened powered infant formula; opened water container	Breast pump parts
WGS analysis summary	Patient isolate (CSF) matched one strain identified in powdered formula	Patient isolates (CSF and blood) matched strain from breast pump
	Isolate from opened water container matched a separate strain identified in powdered formula	
NCBI BioProject number	PRJNA420465	PRJNA420465
Biosample accession number	SAMN26725422	SAMN26725423
	SAMN26725454	SAMN29506921
		SAMN26725456
		SAMN26725453
		SAMN26725453
		SAMN29506919
		SAMN29506919

**Abbreviations**: CSF = cerebrospinal fluid; NCBI = National Center for Biotechnology Information; WGS = whole genome sequencing.

A *C. sakazakii* isolate from the CSF was analyzed by WGS. Samples of opened powdered formula and an opened bottled water container used for formula preparation in the patient’s home were cultured in gram-negative enrichment broth followed by selective media. *C. sakazakii* isolates were recovered from both the powdered formula and the water container. WGS* was performed and identified two distinct strains of *C. sakazakii* (Figure). Sequence reads were cleaned,^†^ assembled,^§^ and analyzed using high-quality single nucleotide polymorphism (SNP) analysis^¶^; results showed that the patient isolate was closely genetically related to an isolate from the powdered formula (0 SNPs apart).** A second isolate from the same can of powdered formula was closely genetically related to an isolate from the water container (within 4 SNPs).^††^ These two strains were not related to one another (>50,000 SNPs apart). Federal partners tested unopened powdered formula from the same lot as the powdered formula consumed by the patient; *C. sakazakii* was not detected.

**FIGURE:**
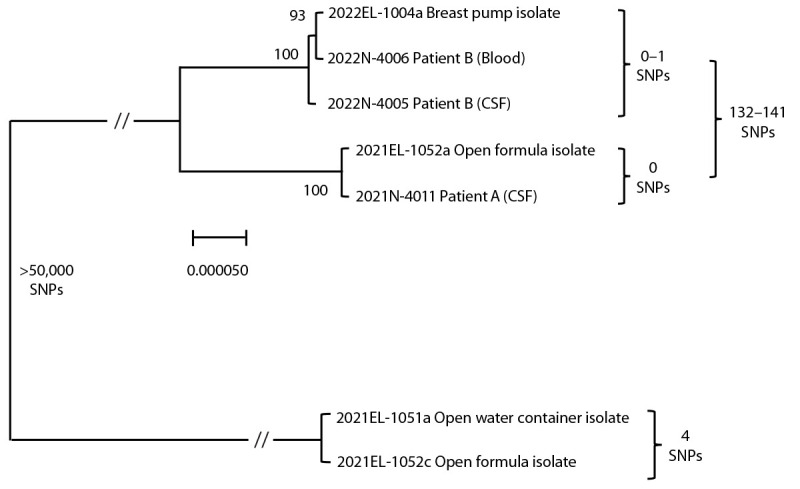
Maximum likelihood phylogeny from high quality single nucleotide polymorphism* analysis^†,§,¶^ of *Cronobacter sakazakii* patient A and B isolates and environmental isolates — United States, 2021 and 2022 **Abbreviations**: CSF = cerebrospinal fluid; SNP = single nucleotide polymorphism; // = skip mark. *SNP distances guide genetic relatedness between strains. ^†^ Scale bar indicates the genomic distance of samples to right of skip mark. ^§^ Values at branch nodes indicate bootstrapping from 100 iterations. *C. sakazakii *isolates from patients A and B, in addition to environmental samples, form three distinct clusters in a maximum likelihood phylogeny. ^¶ ^Skip mark indicates a truncation to capture the relationship between clusters.

The second case occurred in February 2022 in a preterm (gestation of 30 weeks, 6 days), hospitalized male infant (patient B) born by cesarean delivery because of breech presentation and the mother’s worsening preeclampsia. At the time of illness onset, he was being treated in the neonatal intensive care unit for complications of prematurity but was stable, feeding, growing, and breathing without respiratory support. He was fed expressed breast milk fortified with liquid human milk fortifier primarily through an orogastric tube before becoming ill. At age 20 days, he experienced apneic and bradycardic episodes, temperature elevation, and a requirement for respiratory support. Seizures developed the following day. *C. sakazakii* was isolated from blood and CSF cultures. Despite treatment with intravenous antibiotics and repeat negative blood cultures, the patient died 13 days after illness onset.

Expressed milk samples, breast pump parts from two separate devices (one used in the hospital and one used in the mother’s home), and liquid human milk fortifier samples from three lots were cultured in gram-negative enrichment broth followed by selective media; *C. sakazakii* was recovered from the breast pump parts used in the home. An interview revealed that these breast pump parts were cleaned in a household sink, sanitized, and sometimes assembled while still moist. No bacteria were recovered from the expressed milk samples, liquid human milk fortifier samples, hospital breast pump parts, or unopened powdered formula from the hospital. *C. sakazakii* isolates from the patient’s blood and CSF, and those isolated from environmental samples, were analyzed by WGS,^§§^ which indicated that the patient’s CSF and blood isolates were closely genetically related to the isolate recovered from the home breast pump (0–1 SNPs).

## Discussion

On the basis of the WGS analysis, these two cases of *C. sakazakii* infection in infants were not related. One case was likely transmitted by powdered formula prepared in the home, and the other through expressed milk contaminated by breast pump equipment. These cases illustrate the ubiquity of the pathogen in the environment, the importance of hygiene in preventing *C. sakazakii* infections, and the utility of WGS as a method for determining genetic relatedness and probable transmission sources. Understanding more about the sources of these infections can help educate clinicians and caregivers about ways to prevent *C. sakazakii* infections among infants.

*C. sakazakii* can cause meningitis and sepsis, most commonly in very young infants and those with a history of prematurity, possibly because of immaturity of their immune systems and gastrointestinal tracts (*7*). Although *C. sakazakii* infections are treatable with antibiotics, they often have devastating outcomes, with death occurring in nearly 40% of infants who develop meningitis (*2*). Many surviving infants also experience complications, including cerebral abscess and hydrocephalus, which can result in permanent neurologic sequelae (*2*). Because *C. sakazakii* infection is not a nationally notifiable condition, the actual incidence is unknown. However, it is estimated that approximately 18 cases of invasive *C. sakazakii* infection in infants occur annually in the United States, most of which are not associated with outbreaks but likely occur because of isolated instances of contamination of infant feeding products and equipment in the home (*2*,*8*).

Previous investigations have identified *C. sakazakii* in opened powdered formula because of its notable ability to survive in dry environments. However, it has also been recovered from many other environmental sources in the home, including kitchen sink surfaces, pacifiers, bottles, household utensils, vacuum cleaning bags, and other foods (*1**,**2*). Feeding utensils, such as scoops used for powdered formula, can become contaminated on countertops or in sinks and subsequently transfer *C. sakazakii* when reintroduced into the formula. Additional reports have linked *C. sakazakii* infections to contaminated expressed breast milk after recovering the organism from breast pump equipment (*3**–**5*). Phenotypic methods of identification and pulsed-field gel electrophoresis have previously been used to identify *Cronobacter* species and determine potential sources of transmission in cases of infant *C. sakazakii* infections in the United States. However, these methods were limited and could not definitively identify genetic relatedness. Analysis of WGS data from these two cases permitted more accurate determinations of relatedness among isolates. In future investigations, WGS can be used to guide public health practitioners and clinicians about potential outbreaks or sources of *C. sakazakii* related to illnesses.

The findings in this report are subject to at least three limitations. First, *C. sakazakii* infections are not nationally notifiable or reportable in most states, so associations with other strains and detection of potential outbreaks is currently limited. Second, standardized methods for identifying and characterizing *C. sakazakii* are not yet routine, further limiting wide-scale detection and surveillance efforts. Finally, on-site investigations were not performed, so the samples tested might not represent all potential sources of contamination (e.g., additional feeding products, equipment, or environmental surfaces).

Because of the widespread presence of *C. sakazakii* in the environment, caregivers of infants should follow safe hygiene, preparation, and storage practices, and learn steps to protect infants from infection. Clinicians providing care for infants aged <2 months or those who were born prematurely or are immunosuppressed should explain the risks of *C. sakazakii* infection to caregivers, especially if the infant is fed with powdered formula or expressed milk. Education should emphasize exploring alternatives to powdered formula for infants at highest risk and safe powdered formula preparation and storage (*9*). In addition, caretakers should be instructed to thoroughly clean and sanitize breast pump equipment (*10*). Hospitals caring for premature or critically ill infants might consider providing instructions and a dedicated basin for cleaning supplies at home upon hospital discharge to minimize the risk of contamination. Increased awareness of safe hygiene, preparation, and storage practices related to infant feeding products, enhanced understanding of *C. sakazakii* reservoirs, and ongoing public health messaging can help prevent infant *C. sakazakii* infections, complications, and deaths.

**Summary**

**What is already known about this topic?**

Infections caused by *Cronobacter sakazakii* are rare but can cause severe illness and death in infants.

**What is added by this report?**

Whole genome sequencing analysis was used to link one case of *Cronobacter sakazakii* infection in a full-term infant to an opened can of powdered infant formula, and another unrelated fatal case in a premature infant to contaminated breast pump equipment.

**What are the implications for public health practice?**

Increased awareness of the widespread presence of *Cronobacter* in the environment, along with promotion of safe preparation and storage of powdered infant formula, and careful cleaning and sanitization of breast pump equipment, could prevent potentially devastating infections.

## Footnotes

* Genomic DNA was extracted and sequenced on MiSeq instruments (Illumina) following Nextera XT or DNA Prep library preparation.

† Sequence reads were cleaned using run_assembly_trumClean.pl. https://github.com/lskatz/CG-Pipeline.

§ Cleaned reads were assembled using SPAdes (version 3.14.0) https://github.com/ablab/spades.

¶ Lyve-SET (version 1.1.4f) was used for high-quality SNP analysis on reads https://github.com/lskatz/lyve-SET.

** *C. sakazakii* ATCC 29544 was used as the reference genome.

†† *C. sakazakii* isolated from the open formula (2021EL-1052c) was used as the reference genome.

§§ Methods for WGS analysis were identical to the investigation for patient A.
